# From individual to collective 3D cancer dissemination: roles of collagen concentration and TGF-β

**DOI:** 10.1038/s41598-018-30683-4

**Published:** 2018-08-24

**Authors:** J. Plou, Y. Juste-Lanas, V. Olivares, C. del Amo, C. Borau, J. M. García-Aznar

**Affiliations:** Multiscale in Mechanical and Biological Engineering, Aragon Institute of Engineering Research (I3A), Department of Mechanical Engineering, University of Zaragoza, 50018 Zaragoza, Spain

## Abstract

Cancer cells have the ability to migrate from the primary (original) site to other places in the body. The extracellular matrix affects cancer cell migratory capacity and has been correlated with tissue-specific spreading patterns. However, how the matrix orchestrates these behaviors remains unclear. Here, we investigated how both higher collagen concentrations and TGF-β regulate the formation of H1299 cell (a non-small cell lung cancer cell line) spheroids within 3D collagen-based matrices and promote cancer cell invasive capacity. We show that at low collagen concentrations, tumor cells move individually and have moderate invasive capacity, whereas when the collagen concentration is increased, the formation of cell clusters is promoted. In addition, when the concentration of TGF-β in the microenvironment is lower, most of the clusters are aggregates of cancer cells with a spheroid-like morphology and poor migratory capacity. In contrast, higher concentrations of TGF-β induced the formation of clusters with a notably higher invasive capacity, resulting in clear strand-like collective cell migration. Our results show that the concentration of the extracellular matrix is a key regulator of the formation of tumor clusters that affects their development and growth. In addition, chemical factors create a microenvironment that promotes the transformation of idle tumor clusters into very active, invasive tumor structures. These results collectively demonstrate the relevant regulatory role of the mechano-chemical microenvironment in leading the preferential metastasis of tumor cells to specific tissues with high collagen concentrations and TFG-β activity.

## Introduction

The seed and soil theory^1^ was proposed in the late 19th century and has greatly influenced cancer research. Cancer cells represent *the seed*, and their interactions with the microenvironment representing *the soil*, and this relationship was proposed as the main scheme to explain the ability of cancer to spread from its primary location to metastasize in secondary tissues. This theory attempted to explain why cancer does not metastasize randomly and instead exhibits a tissue-specific spreading pattern (tropism) dependent on the type of cancer. Numerous previous studies have focused on the roles of different microenvironmental characteristics and how they affect tropism and cancer dissemination^2–6^, but their results have not explained why different cancers exhibit different modes of migration.

Within this field, the involvement of the extracellular matrix (ECM) as a predictive biomarker of metastasis has recently attracted interest. ECM components are known to play significant regulatory roles in primary tumors by, for example, triggering tumor cell behaviors and invasion^7–11^. In particular, the roles of ECM mechanics in the regulation of cancer cell migration^12,13^ and malignancy development^14,15^ have been extensively explored. However, much less research has been focused on factors that affect the second site of future metastasis. Cell-ECM interactions and physical forces are known to play roles in guiding metastasis^16–18^, but the links between matrix properties and metastasis risk have not been fully explored.

Once circulating tumor cells reach a host tissue, they must adapt to the new microenvironment in order to survive. Their surveillance and proliferation activities are influenced by cell-cell and cell-matrix interactions in the metastatic niche^19,20^. The mechanical properties of the new soil tissue will impact the metastatic behavior of the infiltrating cells. The ECM of the metastatic niche is not simply a passive receiver of circulating tumor cells, its properties, including its composition, stiffness and pore size, provide feedback that affects the behavior of tumor cells. The resulting cross-talk may contribute to whether metastatic colonization is sustained^21^.

Collagen type I (ColI) is the main component of the ECM, and it constitutes the scaffold of the tumor microenvironment. Hence, the amount of ColI in the host tissue appears to be the ECM-related factor that is most important to generating the biochemical forces that form a supportive niche^22^. Changes in collagen concentrations could play a significant role in promoting the survival and growth of distant metastases and may support some zone-specific tumor spread^23^. Furthermore, ColI matrices are abundantly embedded with growth factors and cytokines, such as transforming growth factor-β (TGF-β), which can be released and activated during secondary site tumor colonization.

Therefore, in this study, we aimed to deepen our understanding of the mechanobiological factors that regulate this process and explore how differences in ColI concentrations and TGF-β stimulation directly regulate the key steps involved in metastasis. Here, we recreated and analyzed the behaviors exhibited by metastatic single cells once they reached secondary tissues. We exposed the cells to different collagen concentrations and TGF-β levels to study how these factors and related bio-chemo-mechanical characteristics modulate tumor colonization and surveillance.

For this analysis, we developed a 3D collagen matrix and microfluidics-based cell cultures to quantify the plasticity of the migratory capacity of metastatic non-small cell lung cancer (NSCLC) cells (H1299 cells) and evaluate how cell behaviors depend on the collagen concentration and the presence of TGF-β. Previous studies have shown that 3D *in vitro* cancer models provide numerous advantages over 2D models in studies aimed at exploring cancer malignancy^24–26^. For example, the differential matrix density responses of cancer cell lines have been correlated with tissue tropism^27^. Therefore, we used a simple collagen 3D culture system model to recreate metastatic conditions, and this allowed us to use live-cell microscopy to easily analyze differences in cell migration patterns.

In particular, we found that the collagen concentration in the 3D matrix and the level of TGF-β activation regulated differentiated patterns of cell migration and affected whether cells underwent individual or collective migration. Our results support the notion that the mechano-chemo-biological characteristics of the host tissue play crucial roles in determining the invasive capacity of tumor cells during metastasis.

## Results

### Higher collagen concentrations reduce cancer cell migration

In this study, we sought to mimic the first key steps of tumor cell colonization to explore how different ECM environments affect cancer cell extravasation. First, we tested whether the migration of metastatic NSCLC cells was affected by different collagen concentrations. NCl-H1299 cells were selected as the model for metastatic cells in this study.

Cells were seeded in hydrogels with different concentrations of ColI (2.5, 4 and 6 mg/mL). These collagen-based hydrogels were mechanically characterized in a previous study^28^. Here, we quantified their architecture (Table 1) and found that the stiffness, pore size and porosity of the *in vitro* collagen matrices were similar to the ranges found in various living tissues^12,29,30^.

**Table 1:** Mean pore size (μm), porosity (%) and storage shear modulus (Pa)^28^ of collagen hydrogels with different collagen concentrations after polymerization.

MEAN ± STD	2.5 mg/ml	4 mg/ml	6 mg/ml
Pore Size (μm)	1.97 ± 0.39	1.25 ± 0.32	0.69 ± 0.05
Porosity (%)	99.44 ± 0.29	95.34 ± 2.22	90.53 ± 0.93
Storage shear modulus (Pa)	62.14 ± 4.87	121.03 ± 9.94	254.05 ± 29.06

**Figure 1:**
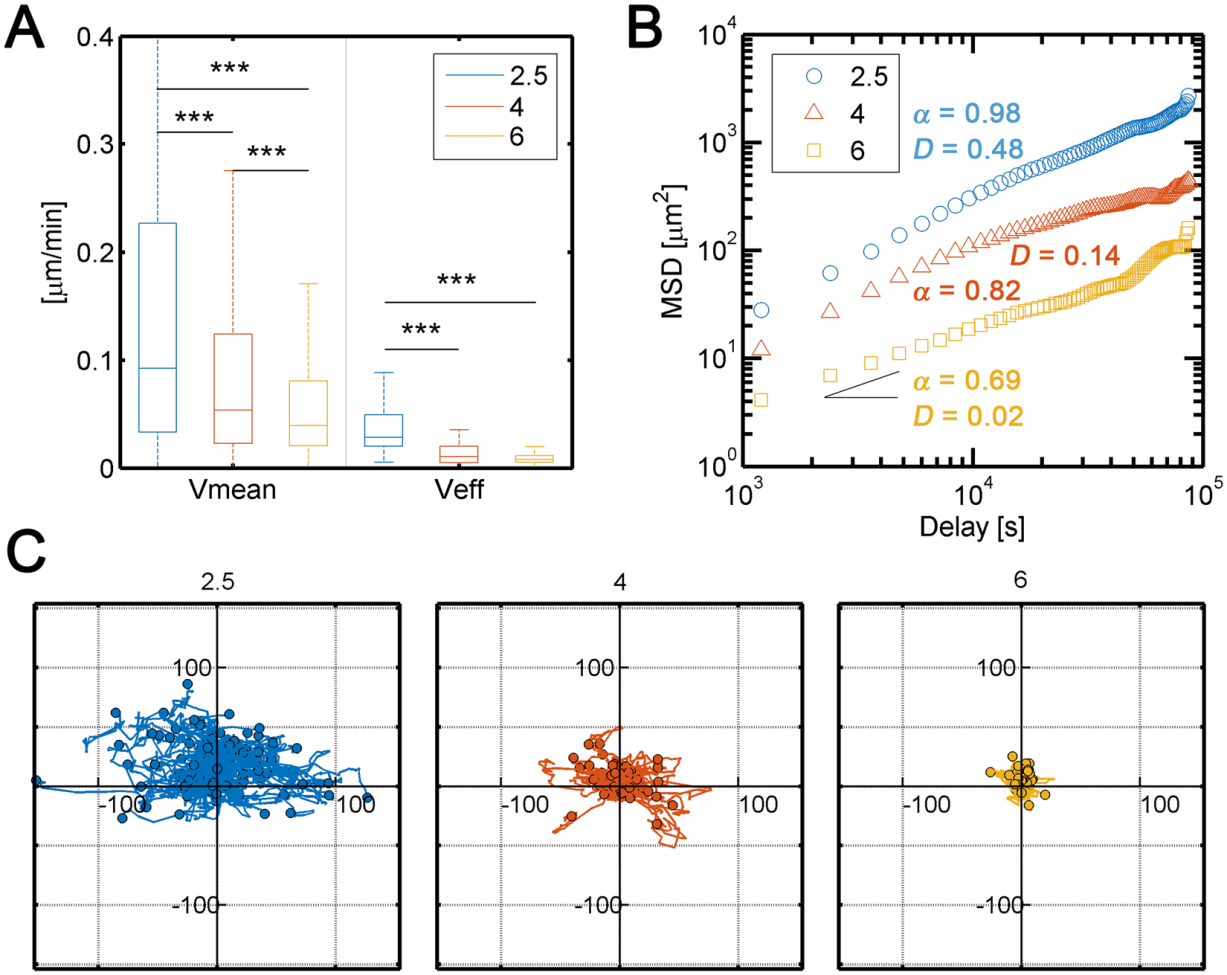
(**A**) Mean (left) and effective (right) speeds of H1299 cells tracked for 24–48 h in different collagen concentrations (2.5, 4 and 6 mg/mL). Both mean and effective speeds significantly decreased as the collagen concentration increased. (**B**) Mean squared displacement (MSD) of the tracked trajectories. Cell migration followed a Brownian motion (*α* ~ 1) pattern, and the diffusive coefficient as higher (indicating higher migratory persistence) (0.48 μm^2^/min) for 2.5 mg/mL collagen. However, cell migration followed a confined motion (*α* < 1) pattern with a lower diffusive coefficient (indicating lower migratory persistence) at higher concentrations (0.14 and 0.02 μm^2^/min for 4 and 6 mg/mL collagen concentrations, respectively). (**C**) Representations of relative cell trajectories. Cells travelled longer distances in lower collagen concentrations. Each condition comprised three repetitions (n = 3). ANOVA followed by post-hoc Tukey-Kramer tests were performed to determine statistical significance. ***p < 0.001; **p < 0.01; *p < 0.05. Statistical data can be consulted in Table 2.

After cell harvesting, the cells were tracked for 24–48 h. The mean (instantaneous) and effective (accounting for only the starting and final positions) speeds were calculated under the indicated conditions. As the collagen concentration increased, the speed of cell migration (both mean and effective) (Fig. 1A) and the diffusion coefficient (as a measure of migration persistence) decreased (Fig. 1B), as expected^13,30^. In fact, we found that there the significant differences observed in cell speed were dependent on the collagen concentration (see Supplementary Videos #1, #2, and #3). This finding was not surprising because collagen density affects the strength of the physical barrier that interferes with cell migration by trapping single metastatic NSCLC cells to prevent their dissemination through the matrix. This phenomenon is known as steric hindrance^31^. Hence, cells become strongly confined when they encounter barriers with higher collagen concentrations, which make migration more arduous, ultimately leading to shorter cell trajectories (Fig. 1C).

**Table 2:** Statistical data of mean and effective speeds for different collagen concentrations with and without TGF-β.

Condition	Vmean							
	N	median	mean	se	∆ mean	CI	p	∆ mean (%)
2.5 C	5181	0.0922	0.1696	0.002	−0.0398	±0.007	0	−23%
2.5 TGF-β	10712	0.0797	0.1298	0.0014				
4 C	3807	0.0537	0.104	0.0023	0.0099	±0.009	0.014	+9.5%
4 TGF-β	5670	0.0695	0.1139	0.0019				
6 C	3837	0.0392	0.063	0.0023	0.0061	±0.009	0.315	+9.7%
6 TGF-β	6231	0.0418	0.0691	0.0018				
	Veff							
2.5 C	82	0,0285	0,0365	0,002	−0,0145	±0,0069	0	−39.7%
2.5 TGF-β	172	0,0145	0,022	0,0014				
4 C	57	0,0105	0,0136	0,0024	0,0042	±0,0088	0,0046	30.9%
4 TGF-β	89	0,0122	0,0178	0,0019				
6 C	60	0,0079	0,0086	0,0024	0,0004	±0,0086	1	4.6%
6 TGF-β	92	0,0063	0,009	0,0019				

A multivariate ANOVA was performed to test both collagen concentration and factor (TGF-β) presence. A Tukey-Kramer post-hoc test was followed to test pairwise significance. From the pairwise comparisons, only those sharing collagen concentration are shown. The full pairwise comparison is summarized in Supplementary Table 1 and graphically plotted in Figs Sup. 2, 3. N: stands for the total number of data points coming from 3 different repetitions of each condition (n = 3); se: standard error of the mean; CI: confidence interval of the mean differences, p: p-value indicating significance.

### Increasing the collagen concentration enhances cluster formation

**Figure 2:**
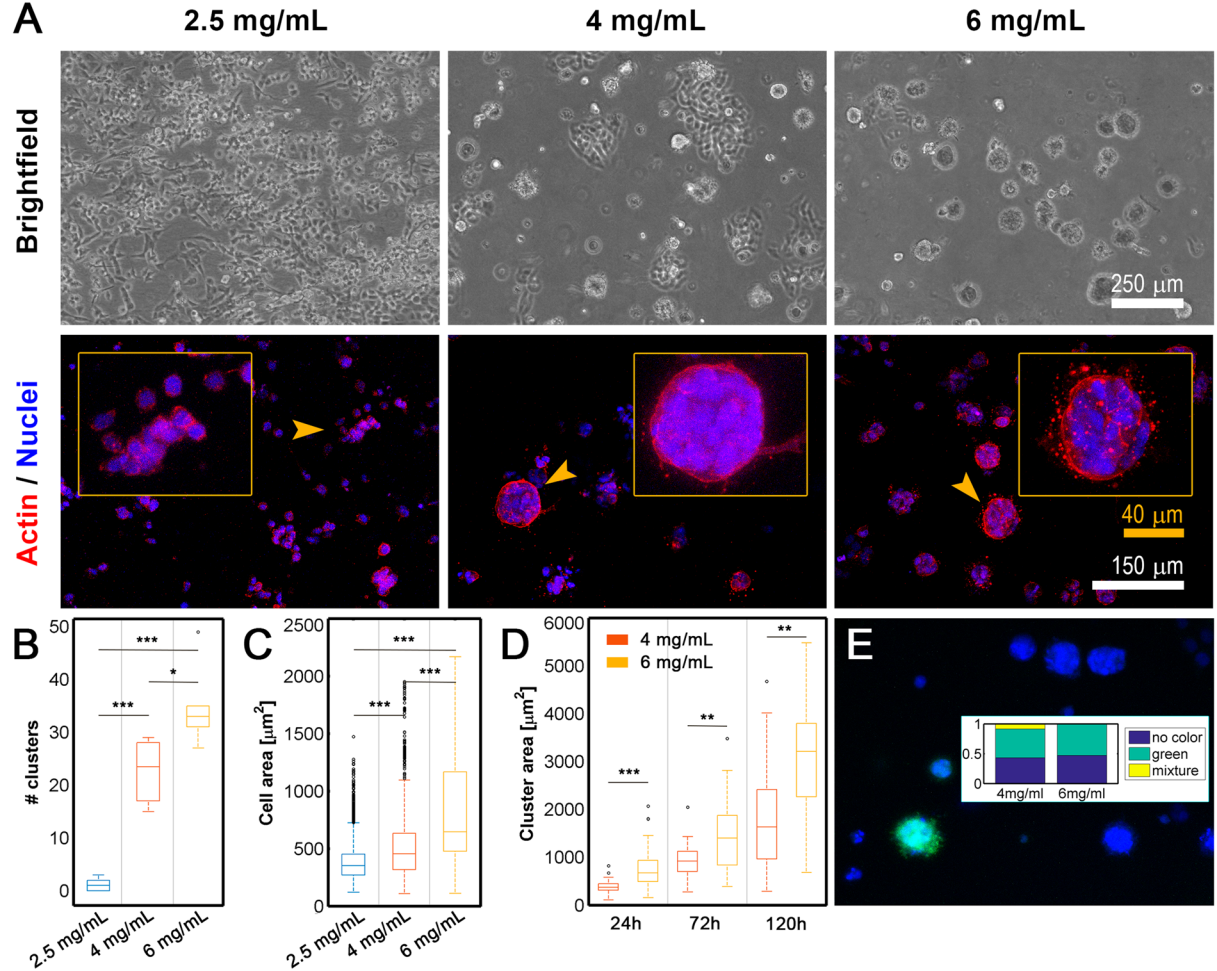
(**A**) Bright-field (scale bar of 250 μm, 100x magnification) and fluorescent confocal (scale bar of 150 μm, 100x magnification) images of H1299 cells after 5 days of growth in the indicated collagen matrices. Yellow arrows indicate the area magnified in the insets (scale bars are 250 μm, 250x magnification). (**B**) Quantification of the number of clusters per device for each collagen concentration (2.5, 4 and 6 mg/mL). Cell aggregates with an area larger than 1000 μm^2^ were considered clusters, and out-of-focus cells were not quantified. Six replicates were performed for each condition. (**C**) Individual cell areas (μm^2^) measured 24 h after cells were seeded in different collagen concentrations (2.5, 4 and 6 mg/mL). The data shown represent the average of the results of 3 replicates for each condition. (**D**) Individual cells and the evolution of cluster area over 5 days in cells grown in 4 and 6 mg/mL collagen matrices. Cluster size markedly increased over time in both conditions. In softer matrices (2.5 mg/mL), no significant growth was observed). (**E**) Confocal images of monoclonal GFP-fluorescent (cyan) and non-fluorescent DAPI-stained clusters that spontaneously formed in 6 mg/mL collagen. The inset shows the percentages of monoclonal (green and no color) and polyclonal (mixture) cell aggregates in the total amount of clusters for cell grown in 4 and 6 mg/mL collagen concentrations. Each condition comprised three repetitions (n = 3). For B and C, ANOVA followed by post-hoc Tukey-Kramer tests were performed to determine statistical significance. For D, Welch tests (a previous Levene test revealed unequal variances) were performed to determine statistical significance. ***p < 0.001; **p < 0.01; *p < 0.05. Each condition comprised three repetitions (n = 3) in all cases (B,C,D).

After 5 days in 3D cell culture, single H1299 cells were able to proliferate under all conditions. However, different matrix-dependent morphologies were observed (Fig. 2A). Multicellular clusters of cells spontaneously formed in higher collagen concentrations (4 mg/mL and 6 mg/mL) and showed a rounded morphology with multiple DAPI-stained nuclei, whereas in 2.5 mg/mL collagen matrices, spreading single cells were mainly observed. In fact, as shown in Fig. 1C, cells were more capable of dissemination through the 2.5 mg/mL matrix, resulting in the formation of fewer clusters.

In contrast, as the collagen content increased, cluster formation increased, the proportion of single cells markedly decreased. Significant differences in the number of growing clusters were observed among the conditions. As shown in Fig. 2B, when the average number of clusters per microdevice was determined, the results indicated that very few clusters formed in softer matrices (1.15/device), whereas up to 23.5 and 33 clusters/device were counted in the 4 mg/mL and 6 mg/mL collagen concentrations, respectively. In all cases, we defined multicellular clusters as cell aggregates with an area larger than 1000 μm^2^.

### H1299 spheroid growth is dependent on collagen gel concentration

We measured individual cell areas at different collagen concentrations at 24 h after seeding. Cluster growth was then monitored over 5 days.

On the first day, individual cell areas were larger in cells grown in higher collagen concentrations (Fig. 2C), in agreement with previous findings showing that malignant epithelial cells tend to occupy larger surface areas when grown on stiffer matrices^15,32^.

Cell proliferation caused the average area of the tumor clusters to grow dramatically over time in the 4 mg/mL (from ~500 to ~3000 μm^2^) and 6 mg/mL (from ~1000 to ~3750 μm^2^) collagen matrices (Fig. 2D), whereas the cells grown in softer matrices (2.5 mg/mL) did not tend to form clusters or increase cluster size.

The proliferative ability of H1299 cells enables these cells to form viable monoclonal clusters that start with single cells. By harvesting GFP-transfected H1299 cells together with non-fluorescent H1299 cells, we show that two types of predominant cell clusters develop: fluorescent and non-fluorescent ones. The general lack of mixed clusters demonstrates the monoclonal character of the clusters and implies that each cluster formed from a single initial cell (Fig. 2E).

### TGF-β promotes cluster motility and a stretched morphology

We further analyzed the impact of epithelial cell TGF-β on the migration of single cells and cluster formation. TGF-β plays a dual role in human cancer by acting as either a tumor suppressor or a promoter of tumor metastasis^33^ depending on a variety of factors, such as the state of the disease^34^ and matrix rigidity^35^. Once local metastases take hold, local production of TGF-β can profoundly affect the growth of these lesions. The release of TGF-β in the vicinity of a secondary tumor is correlated with metastatic colony expansion and tumor reinitiation^36,37^. We sought to determine how TGF-β affects tumor cell migration in cells grown in different collagen concentrations and for different incubation times.

**Figure 3:**
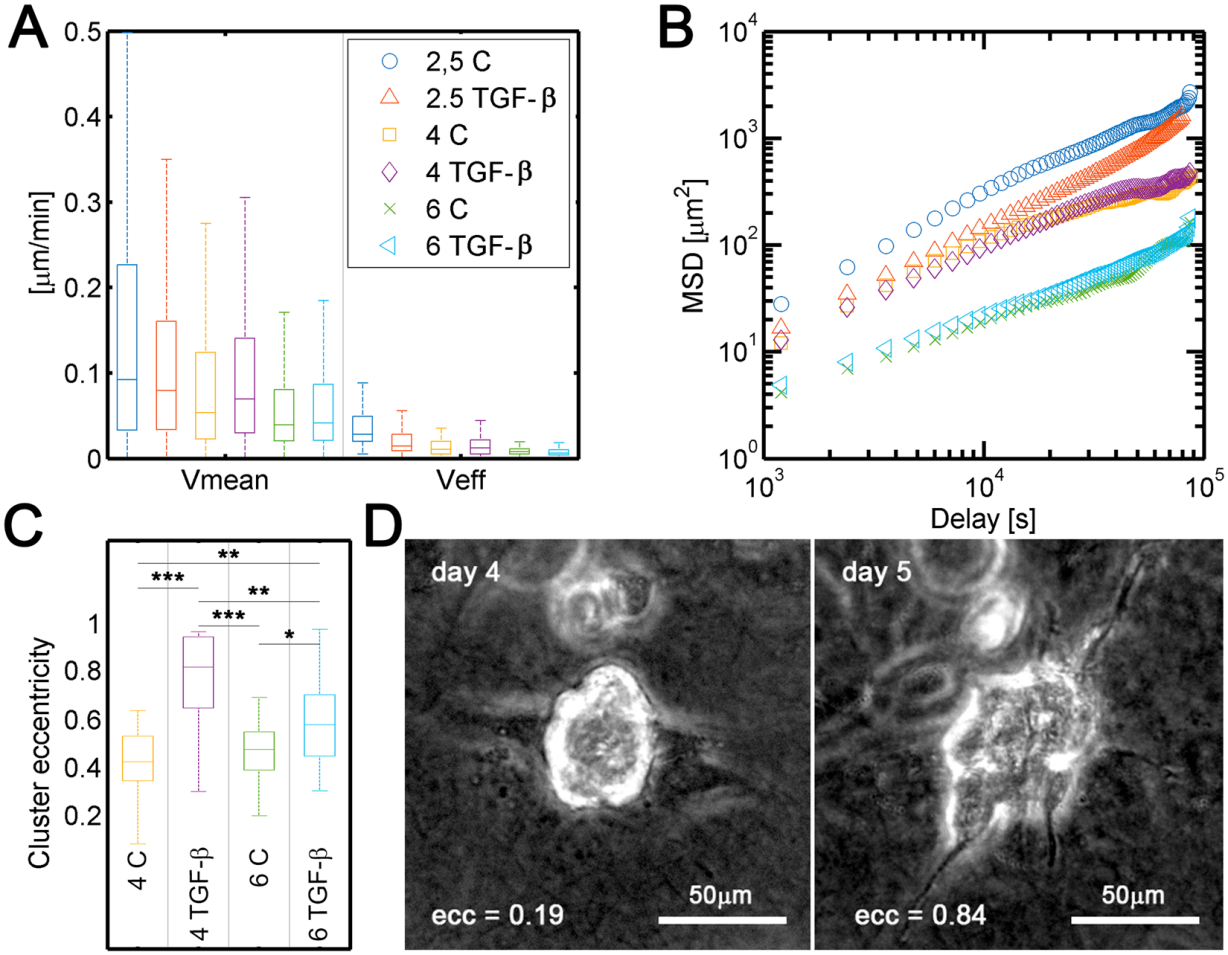
(**A**) Mean and effective speeds of H1299 cells (grown with or without TGF-β and at different collagen concentrations (2.5, 4, and 6 mg/mL), cumulative density function of the mean velocities are represented in Fig. Sup. 1. (**B**) Mean squared displacement (MSD) of tracked trajectories. TGF-B decreased cell diffusion (migration persistence) on softer matrices (2.5 mg/mL) but had smaller influence on stiffer matrices. (**C**) Cluster eccentricity at day 7 in cells grown under control and activated conditions with TGF-β in different collagen concentrations (4 and 6 mg/mL). Each condition comprised three repetitions (n = 3). A multivariate ANOVA followed by post-hoc Tukey-Kramer pairwise tests were performed to determine statistical significance in A and C (*markers in A are not shown for clarity). ***p < 0.001; **p < 0.01; *p < 0.05. Numerical statistical data of A can be consulted in Table 2 and in Supplementary Table 1. (**D**) Bright field images of the same cluster at different times. The eccentricity value increases from 0.19 at 4th day, when it was still considered as a spheroid (eccentricity < 0.8) to 0.84 at day 5th when it turned into a strand (eccentricity > 0.8).

The cells were initially pre-treated with TGF-β for 3 days in 24-well plates before they were harvested for the 3D model. Then, individual cell migration was tracked. During the first 48 h, some differences were observed between pre-activated and non-activated individual cells in both migration speed and persistence (Fig. 3A,B). TGF-β seemed to have an effect on individual cell migration and its functional response may change in relation to matrix rigidity^35,38,39^. In fact, a multivariate statistical analysis revealed a strong interaction between collagen concentration and the presence of TGF-β (Fig. Sup. 3). With the addition of TGF-β, mean cell speed significantly decreased (−23%) at lower collagen concentrations (2.5 mg/ml), but increased or remained the same in denser matrices (4 and 6 mg/ml respectively). Changes in persistence (effective speed) were even more pronounced, decreasing almost a 40% in 2.5 mg/ml concentrations and rising a 30% in 4 mg/ml gels. Persistence also increased modestly (about 5%) in denser collagens (6 mg/ml) but this effect was statistically non-significant (see Table 2). Treatment with TGF-β seemed to activate molecular pathways that diminish steric hindrance effects at higher collagen concentrations (Fig. 3A). Based on this previous result, we performed another approach to study this phenomenon of interaction between collagen concentration and TGF-β. In this case, cells were cultured in 3D collagen matrices, and TGF-β activation was induced after gel polymerization. After seven days in 3D cell culture with 2.5 mg/mL matrices, the cells treated with TGF-β showed a morphological response and appeared as individual cells with a more elongated phenotype than was observed in the non-activated samples, in agreement with previous studies^40^. At higher collagen concentrations (4 and 6 mg/mL), cluster growth was stimulated and macroscopic changes were observed.

**Figure 4:**
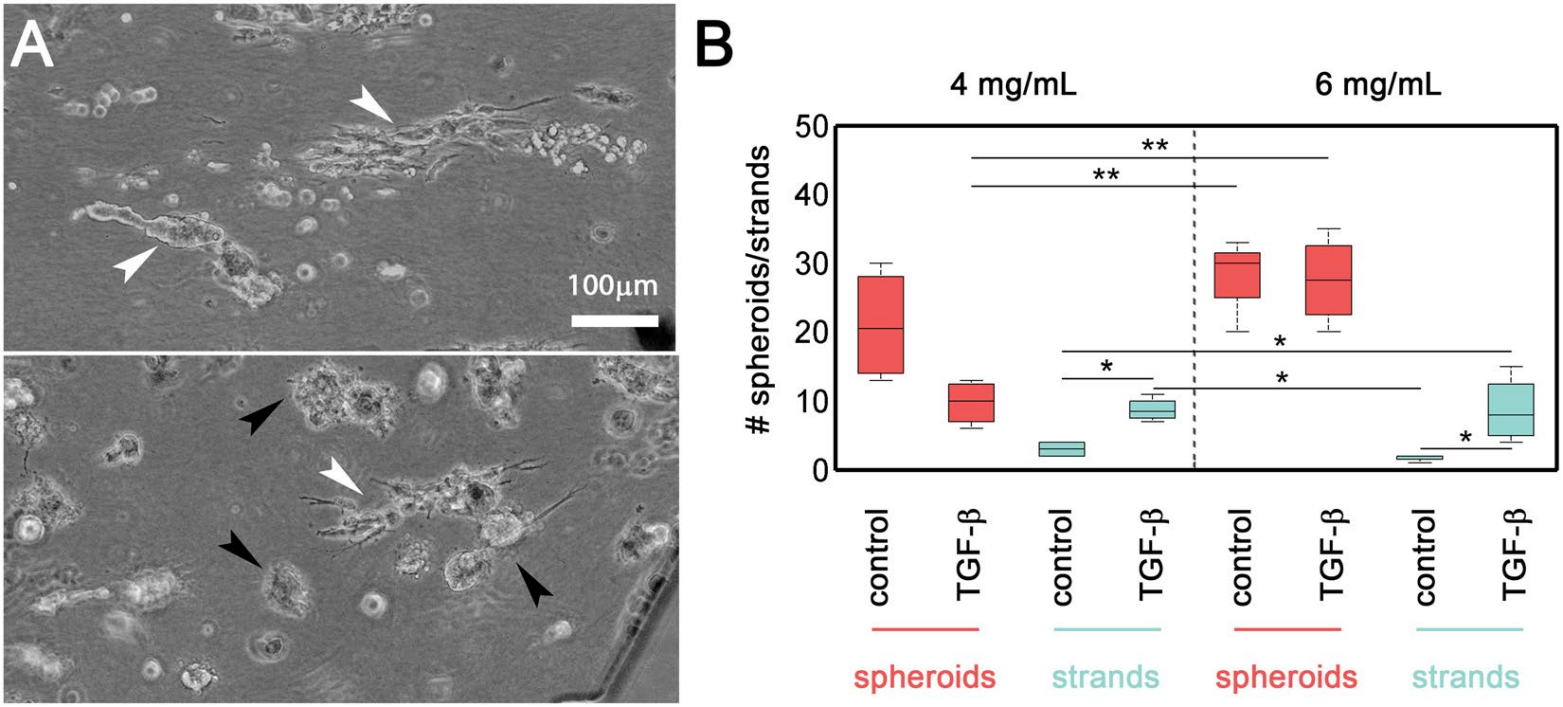
(**A**) Bright-field images taken after 7 days of treatment with TGF-β in cultures grown in 4 mg/mL (left) and 6 mg/mL (right) collagen matrices. Strand-like morphologies (eccentricity > 0.8) are indicated with white arrows. Spheroids are indicated with black arrows. The scale bar is shared by both panels. (**B**) Quantification of the number of strand-like and spheroid-like clusters per device in cultures grown in 4 mg/mL and 6 mg/mL collagen matrices. Each condition combination consisted of 4 replicates. A multivariate ANOVA followed by post-hoc Tukey-Kramer pairwise tests were performed to determine statistical significance (***p < 0.001; **p < 0.01; *p < 0.05). Spheroid and strand data sets were considered separately. The full pairwise comparison is summarized in Supplementary Table 2 and graphically plotted in Figs Sup. 4,5.

We quantified the roundness of the clusters formed at these concentrations by measuring the eccentricity of their occupying areas. We arbitrary defined an eccentricity threshold of 0.8 to differentiate spheroids (<0.8) from strands (>0.8). After cells were incubated for seven days in non-activated 3D cell culture (controls), cell clusters had an overall round morphology. However, when activated with TGF-β, the strand morphology was strongly promoted, especially in the 4 mg/mL collagen matrices (Fig. 4A,B). This effect was associated with a global increase in eccentricity values, which rose from 0.45 to 0.87 in 4 mg/mL collagen and from 0.46 to 0.65 in 6 mg/mL collagen (Fig. 3C,D).

The percentage of cells with a strand morphology increased from 12.5% in the control 4 mg/mL matrices to 45.7% in the TGF-β-activated matrices. There were also significant variations in the 6 mg/mL matrices, in which the strand percentage rose from 5.8% to 24.3% (Fig. 4B). This result suggests that TGF-β signaling induces changes in cell behavior and enhances strand tumor dissemination, at least within this range of collagen concentrations. Indeed, a multivariate statistical analysis revealed that the formation of spheroids is significantly dependent on collagen concentrations (p = 0,0017), while TGF-β determines the evolution to strand-shape clusters (p = 0,0004). In fact, the interaction between collagen and TGF- β to explain the formation of strands, was strongly non-significant (p = 0,6454) (Fig. Sup 5). Hence, these results indicate that TGF-β signaling plays a critical role in regulating collective migratory plasticity^41^.

### Collective migration: migration is faster and more directed in strands than in spheroids

**Figure 5:**
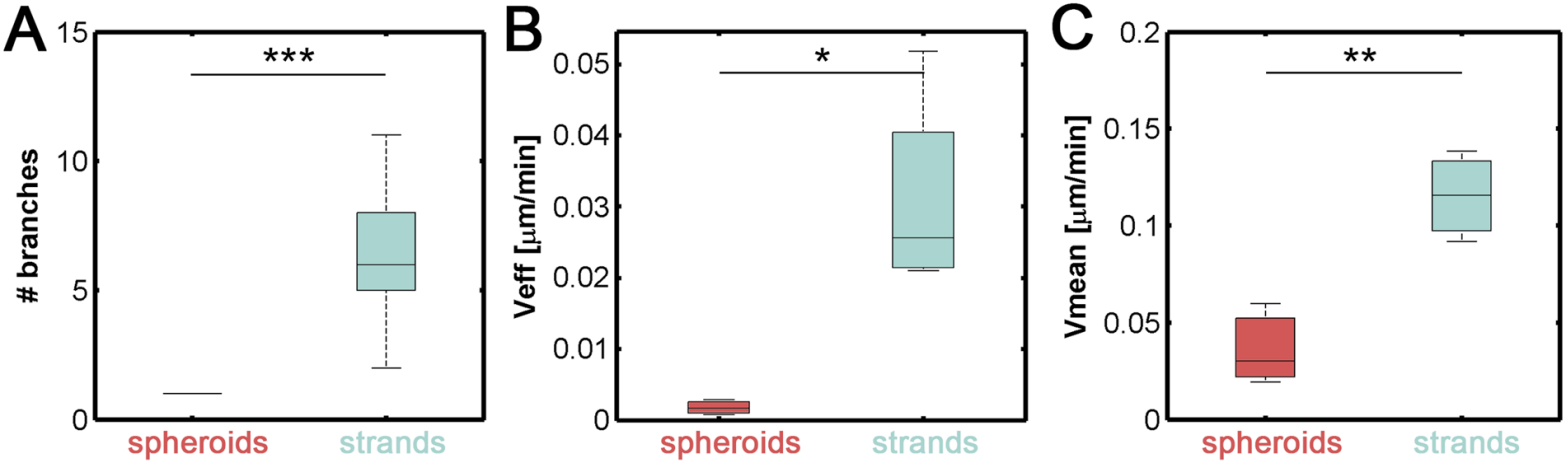
(**A**) Average number of branches generated by spheroid-like and strand-like clusters. (**B**) Mean collective velocity of H1299 cells after 7 days of treatment with TGF-β treatment in cells grown in 4 mg/mL collagen matrices. The presence of leading cells and their elongated morphology allowed the strand-like clusters to move at much higher velocities than were observed for spheroids. (**C**) Effective speeds of H1299 cells. Strand-like clusters migrated in a more directed fashion than was observed for spheroids. All data were collected from 4 independent experiments on the seventh day after the cells were studied for 24 h. For A, ANOVA test was used to determine statistical significance, whereas for B and C (a previous Levene test revealed unequal variances) Welch tests were performed. ***p < 0.001; **p < 0.01; *p < 0.05.

Migration parameters (cell speed, cell displacement and number of branches) were quantified in TGF-β-activated 4 mg/mL collagen matrices. We used bright-field time-lapse microscopy to follow the invasion pattern for 24 h to analyze cancer progression. During this time, the shapes of the spheroids remained stable while developing few or no branches (Fig. 5A), indicating poor invasiveness. Irregular cluster growth shifted the spheroids’ center of gravity, producing wiggling but ineffective movement (see Supplementary Video #4).

Conversely, strand-like clusters were capable of actively migrating through the matrix. We observed these cells acted cooperativity to produce a speed that highly surpassed that observed in slower spheroid migration (Fig. 5C). In fact, the strand-like clusters continued to increase in size while simultaneously developing the capacity to efficiently disseminate through the matrix at an average speed of 0.11 µm/min, which was faster than the 0.029 µm/min observed in cell spheroids. In addition, strand movement was much more directional in strands than in spheroids, as reflected by their significantly different effective speeds (0.025 and 0.0025 µm/min, respectively) (Fig. 5B). This cell behavior was directly related to the number of protruding arms or branches that emerged from the clusters. These branches appeared to lead the cells, and they constantly explored the cluster’s surroundings and sometimes dragged the entire body of the cluster in a specific direction. This dragging, in turn, generated the observed elongated morphology and eventually produced a substantial displacement of the cluster’s center of gravity (see Supplementary Video #5). Therefore, strand-like clusters were more likely to advance in the principal direction, and this enhanced the invasive capacity of these tumor cells.

### Strand formation implies an integral reorganization of cell actin structures

**Figure 6:**
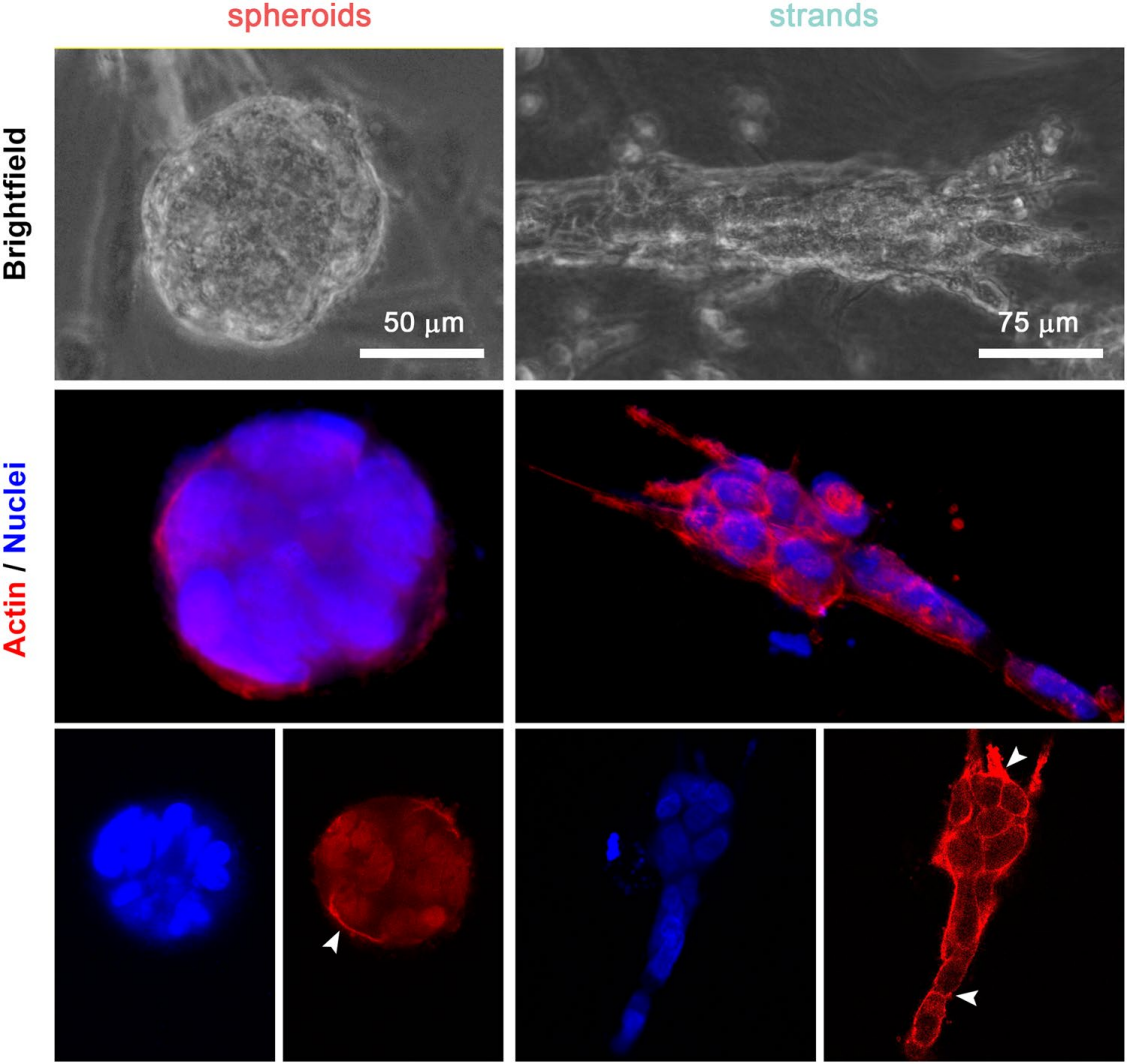
(**A**) Bright-field and 3D reconstructions of confocal images of individual H1299 cell clusters after 7 days of growth in 4 mg/mL matrices. Strand-like clusters are characterized by the presence of actin-filled protrusions, whereas spheroids showed a non-internal actin distribution and a lack of significant protrusions (400x magnification in bright-field pictures and 600x magnification in confocal pictures). Last row shows the color channels (actin: red; nuclei: blue) separately of a 2D focal plane to enhance the visibility of both cellular structures (white arrows point actin focalization zones).

Time-lapse movies and F-actin staining revealed that the movement of clusters in dense collagen matrices evolved from expansive growth movements to migration as strands. This process began at the cluster’s periphery, from which cell protrusions extended, and after a certain degree of structure strengthening was accomplished, these cells eventually escaped from the spheroid into the collagen and then started to pull the cluster’s body along while guiding its movement (see Supplementary Video 6). This reshaped morphology was accompanied by an alteration in F-actin localization. As shown in Fig. 6, actin fibers are clearly visible in cells with a strand morphology, whereas those in spheroids seem to have a focalized distribution pattern in which actin was localized at the cell cluster periphery. In contrast, in EdU assays, there were no significant differences in proliferative rates between spheroids and strand clusters (Fig. Sup. 6), indicating that TGF-β did not affect cell division.

In multicellular cancer invasion, steric hindrance and microtracks with dimensions smaller than the cell diameter (Table 1) caused the cells to become deformed and to aggregate^42,43^. In the cultures activated by TGF-β, the leader cells were located at the invasive front and were characterized by an altered morphology, which might had led to the generation and enlargement of predefined microtracks, eliminating the need for cell followers to generate new tracks^44^. Therefore, the observed intense actin polymerization may have been necessary to achieve harmonized migration.

## Discussion

In the present study, we harvested a metastatic NSCLC cancer cell line in microfluidic devices and varied both the ColI concentration and the presence of TGF-β to explore how ECM conditions play a role in the dissemination of cancer cells and contribute to the first steps of tumor colonization. We established how the microenvironment can regulate the plasticity modes observed in cancer migration, during which cells evolve from single to collective migratory modes, and quantified the dynamic and morphological factors that differed between these modes of invasion. Our initial results supported the hypothesis that a metastatic genotype is not the only parameter that plays an important role in tumor dissemination in all “soils”^45^ and indicate that both the collagen concentration and amount of TGF-β are important modulators of this process.

In a previous study, H1299 cells were inoculated into athymic mice via an intracardiac route, and the authors observed that the frequency of metastasis was higher in bone than in soft tissue^46^. Collagen-rich interstitial matrix is known to decrease cell velocity and consequently tissue invasion. Therefore, the following questions have arisen: why is the colonization of tumor cells (including the H1299 cell line) correlated with tissues with high ECM density, such as bone^47^, which has an ECM more than 10^5^ times more rigid than that found in other matrices in soft tissues^36,48^? What explains the link, which has been reported both *in vivo* and *in vitro*, between desmoplasia (the growth of fibrous or connective tissue) and a higher probability of colonization by tumor cells^49–51^?

Our research results indicate that cancer cells grown in enriched collagen matrices migrate more slowly and tend to spontaneously form cell clusters^22^ and suppress single cell invasion. In dense matrices, cluster formation is induced by the high grade of cell confinement via a phenomenon called cell jamming^52^. The aggregated cell clusters present in metastases play a significant role in metastatic cell surveillance by increasing metastatic propensity and colony formation because they are more resistant to apoptosis at distal metastatic sites^53–56^. Therefore, as observed in this study, cell aggregation and cluster stability are enhanced in denser matrices. There, they are likely to promote cell surveillance during the first steps of colonization, even though they are associated with low invasive ability because the cells remain confined in the matrix region. Furthermore, we propose that using this cancer-on-a-chip system to study the cell-cell junctions that govern this process may offer valuable information, and we intend to use this approach in our future works.

In this work, we attempted to investigate the effects of the cytokines located in these high-density matrices on migration. TGF-β is known to play a major role in orchestrating such processes, including osteoblast-osteoclast signaling in bone remodeling, osteoclastic bone resorption^57^ and the development of fibrosis^58^. In fact, it has been argued that tumor cells embedded in these dense tissues produce factors that favor the increased release of TGF-β^5,36^. Furthermore, inhibiting the TGF-β pathway reduced the formation of bone metastases in preclinical models, and TGF-β seems to be related to the appropriate activation and progression of disseminated tumors^36,37,59,60^. On the other hand, there are numerous works^35,38,39^ which highlight the importance of integrin-TGF-β crosstalk and the manner that this pathway interaction controls the switch in cellular response to TGF- β induced by matrix rigidity, as it was quantified in this manuscript.

We next treated the denser collagen matrices (i.e., 4 and 6 mg/mL) with TGF-β. This treatment promoted the development of strand morphologies after 7 days, especially in cells grown in 4 mg/mL collagen, as previously shown_._ TGF-β signaling is well-known to break the junctional protein localization at the tumor-stromal interface and affect actin cytoskeleton reorganization trough Smad signaling pathway. Some studies have revealed that in the presence of TGF- β, tumors maintain E-cadherin membrane localization in multicellular lobular tumor structures but cytoplasmic localization or potential degradation in peripheral cells, promoting a strand-like migration^42,52,61,62^. This Collective-strand migration offers an effective and more aggressive invasion mode for multicellular units, minimizes the energy costs to individual cells and allows groups of cells to invade further and coordinate their movements with other cells, among other properties^2,63,64^. In this context, we found that the collective cell migration observed in the strand-like clusters grown in 4 mg/mL collagen had higher velocities than were observed in individual cells, even when we accounted for their fastest speeds on softer matrices (0.092 µm/min). This value (0.11 µm/min), which closely approached the velocities observed in *in vivo* intravital imaging experiments^16^, indicates that TGF-β exerted a strong effect by causing cells to switch between a mode encompassing expansive growth with minimum movement to one defined by a strand migration state with high invasiveness. Additionally, mechanical forces exerted by tumor cluster on extracellular microenvironment are likely to play a pivotal role in this migration mode switch induced by TGF-β and need further investigation.

Hence, we show that overt tumor colonization can be recreated under these conditions in microfluidic devices. These results provide a new perspective on the mechanism by which tumor cells overcome steric hindrance in dense matrices. The increased invasiveness and higher survivability of cell clusters may play a role in high collagen-TGF-β matrix tropism. Moreover, future approaches should use a diversity in tumor cell lines so that this hypothesis can be generalized and to unravel the molecular basis of the crosstalk observed between TGF-β and collagen^39^. As a proof of concept, a highly metastatic breast cell line (MDA-MB-231), that produces bone metastases^27^, was also tested under conditions including a high collagen concentration (6 mg/mL) and treatment with TGF-β. We achieved results (see Fig. Sup. 7) very similar to those found in NSCLCs. Therefore, and although this hypothesis should be explored in future experiments performed using many more and varied tumor cell lines, our results support the assumptions that a high collagen concentration regulates cluster growth and that TGF-beta mediates their capacity for invasion.

**Figure 7:**
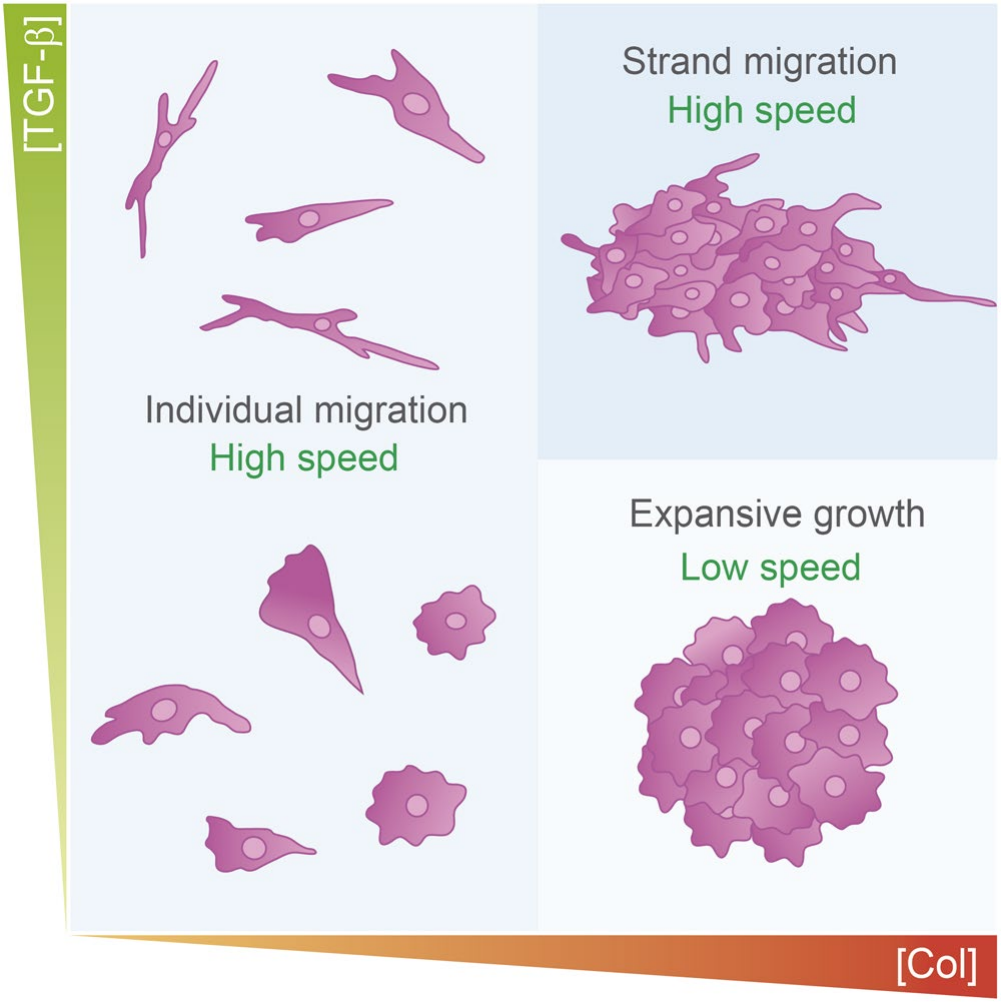
Graphical representation of the different cell morphologies observed in the 3D matrices in this study. At 2.5 mg/mL ColI, individual cell migration predominated (median speed, 0.092 μm/min). Cells aggregated as the collagen concentration increased, resulting in the formation of clusters with very low levels of expansive growth (average, 0.029 μm/min). Cells stimulated with TGF-β and grown in a dense matrix became more aggressive, changed their morphology and started high collective strand migration via leading cells (average velocity, 0.11 μm/min).

In summary, the collagen concentration of biological fiber matrices represents a strong physical limitation on the capacity of cancer cells to migrate and proliferate, suggesting that collagen concentrations regulate the formation and growth of tumor spheroids in metastatic tumors. Thus, a combination including a high collagen concentration and an environment rich in TGF-β will promote the capacity of tumor cells to invade host tissues (Fig. 7).

## Materials and Methods

### Microfluidic platform

Microdevices were used according to the methodology described by Shin *et al*.^65^. Therefore, soft lithography was used to develop positive SU8 240-µm relief patterns with the desired geometry on a silicon wafer (Stanford University). Polydimethylsiloxane (PDMS, Sylargd 184, Dow Corning GmbH) was mixed at a 10:1 weight ratio of base to curing agent. The mixed solution was poured into the SU8 master and then degassed to remove air bubbles. Once the solution was cured, the replica-molded layer was trimmed, perforated and autoclaved. In the final step, the PDMS device and 35-mm glass-bottom petri dishes (Ibidi) were plasma-bonded and treated with poly-D-lysine (PDL) at 1 mg ml^−1^ (Sigma-Aldrich) with the aim of improving surface-matrix attachment.

**Figure 8:**
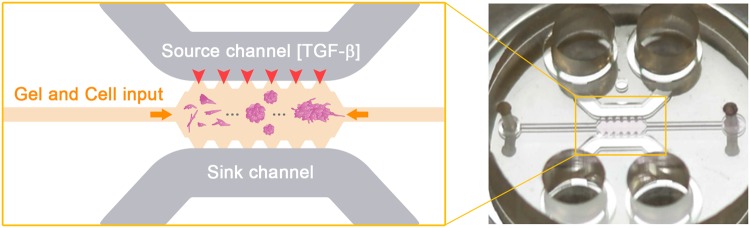
(**A**) Picture of the microfluidic device that was placed inside a 35-mm plate (**B**) Scheme of the central part of the device, which contained a central channel filled with collagen and an embedded cell trough auxiliary channel (orange horizontal arrows). The cells were harvested for 5–7 days. The source and sink channels ensured hydration and the diffusion of TGF-β through the hydrogel.

The device geometry was based on that used by Farahat *et al*.^66^, which contained a central cage into which the hydrogel containing the embedded cells was deposited. In connection with the gel, it also had two side media channels that ensured hydration and the transport of nutrients and other chemical factors throughout the hydrogel (Fig. 8).

### Hydrogel preparation and cell seeding

#### Cell culture

Metastatic NSCLC H1299 is a cell line that was derived from a metastatic site and expresses a variety of metastasis-promoting factors. H1299 cells were cultured using RPMI-1640 (Lonza) medium supplemented with 10% fetal calf serum (FCS, Life technologies). Cells were passaged or used for experiments when they reached 80% of confluence. H1299 cells were seeded into collagen-based hydrogels after sequential trypsinization and centrifugation. Finally, the cells were mixed with gel solutions at a final concentration of 0.2 × 10^6^ cells/mL and pipetted into the gel cavities of the microdevice.

#### Collagen solution

We followed the protocol described by Shin *et al*.^65^ Briefly, ColI (BD bioscience) was buffered to a final concentration of 2 mg/mL, 4 mg/mL, or 6 mg/mL with 10x DPBS containing calcium and magnesium (Thermo Fisher), cell culture-grade water (Lonza), and a previously prepared cell solution. The dilution was brought to pH 7.4 with NaOH.

#### Hydrogel polymerization

Gel scaffold regions were filled with gel solution samples using auxiliary channels. The surface tension caused by the posts enabled the collagen gel solution to fix into the gel cavity. The gel-filled devices were then placed in prepared humid chambers in a CO_2_ incubator to allow the collagen to polymerize at 37 °C for 20 min. After polymerization, the gels were hydrated with RPMI and stored in the incubator for 24 h prior to conducting experiments. This incubation time ensured the stabilization of the matrix and cell adhesion and conditioning. The 3D systems were ready to use at 24 h after polymerization. The culture media (RPMI-1640, 10%FBS) was renewed in both media channels every day, and cells were cultured for 5–7 days.

### Chemical activation: addition of TGF-ß

**Figure 9:**
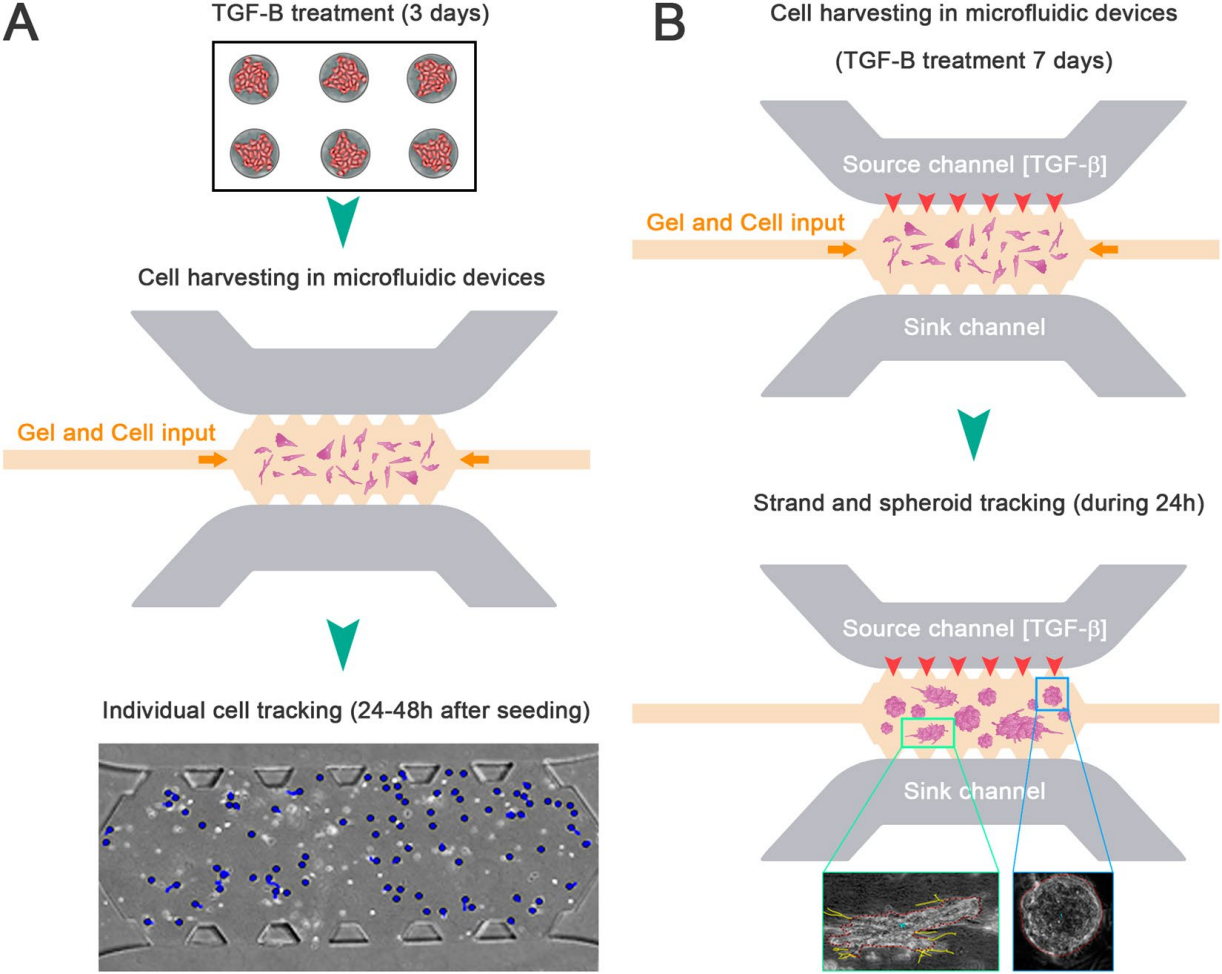
Schematic representation of the two TGF-β activation protocols performed along the manuscript. (**A**) Three days TGF- β treatment in a 24-well cell culture and subsequently activated cells were harvested into the 3D microdevices. The TGF-β effects on individual cells migration was automatically tracked between 24 h and 48 h following cell seeding. (**B**) Direct cell activation with TGF- β in the microfluidic device for 7 days. Cell media with TGF- β (10 ng/ml) was replaced once every day. Finally, cell aggregates were tracked during 24 h.

Different assays were performed to assess the effects of epithelial cell TGF-β (Miltenyi Biotec) on cancer cell migration (Fig. 9). The concentration of the recombinant human TGF-ß isoform employed in these experiments was 10 ng/ml^67^.

In one group (Fig. 9A), the cells were treated with 10 ng/ml of TGF-β for three days in 24-well cell culture plates and subsequently harvested and placed in 3D microdevices. In these experiments, individual cell migration was studied at 24 h after cell seeding.

In a second group (Fig. 9B), the cells were first seeded without previous TGF-β treatment. After the cells were embedded in 3D collagen matrices with different collagen concentrations, they were stimulated with TGF-β for seven days. The cell media containing TGF- β (10 ng/ml) was replaced once every day. Cell morphology and collective migration were subsequently evaluated after seven days.

### Immunofluorescence staining and imaging

The samples were stained with DAPI and phalloidin and imaged using a Nikon D-Eclipse C1 confocal microscope equipped with a Plan Apo VC 60XH objective and an Olympus Fluoview FV10i confocal microscope equipped with an UPLSAPO 60XW objective. The cells were fixed in 4% paraformaldehyde (Affymetrix) in PBS for 20 min at room temperature, washed three times with PBS and permeabilized with 0.1% Triton X-100 (Calbiochem) in PBS. The cells were washed three washes more times and then blocked in 3% goat serum (Sigma) in 5% BSA/PBS solution for 4 h at room temperature. Afterwards, the devices were incubated overnight at 4 °C in the dark with Alexa Fluor® 594-phalloidin conjugates (A12381, Molecular Probes). Then, the samples were washed 3 times with 0.5% BSA/PBS, and the devices were incubated for 4 h at 4 °C in the dark with DAPI (Invitrogen). Finally, the cells were washed 3 more times and imaged.

To study cell proliferation in cell clusters and strands, the cells were cultured with 10 µl of EdU (Merck) per ml of culture medium for 24 h prior to imaging acquisition. The cells were fixed with paraformaldehyde and permeabilized with Triton X-100. The cells were incubated for 30 minutes with the following reaction cocktail for cell staining: 6-FAM Azide, Catalyst solution, and Reaction buffer (Merck).

### Cell tracking and morphology quantification

Time-lapse imaging was carried out by acquiring phase-contrast images every 20 min for 24 h to study individual cell migration. The focal plane located in the middle of the device along the z-axis was selected, and out-of-focus cells were not quantified to minimize artifacts resulting from the glass and PDMS surfaces. This approach ensured that the tracked cells were fully embedded within the 3D network. The incubation conditions were controlled at 37 °C, 5% CO_2_ and 95% humidity. Approximately 50 cells in each set of experimental samples were tracked. Cell trajectory acquisition was performed using a hand-coded semi-automatic MATLAB script described in previous studies^68–70^. By comparing pixel intensities and using matrix convolution techniques, the software was able to find and track cell centroids, request visual correction from the user, and post-process the migration results. These trajectories were used to extract the cell mean (Vmean) and effective (Veff) velocities. Note that Vmean is defined as the averaged instantaneous speed including all time steps, whereas Veff takes into account only the initial and final positions. The MSD curve of each trajectory was also obtained and used to determine the global diffusion coefficient (D), which was used as a measure of migration persistence in a linear-weighted fit of the mean MSD curve (using the first quarter of the data). Additionally, MSD individual curves were used to fit a power law (MSD(t) = γ.tα)) to determine the kind of motion (α < 1 for confined motion, α = 1 for Brownian or purely diffusive motion and α > 1 for directed motion)^71^.

Cluster/strand morphological characteristics were also obtained with a MATLAB hand-coded script. We compared the roundness of formed clusters/strands in different experiments using eccentricity as a measurable scalar parameter that was defined as the ratio of the distance between the foci of the ellipse that fit the cluster/strand (the ellipse with the same second-moments as the region) to its major axis length. The values ranged between 0 and 1, with 0 indicating a circle and 1 indicating a line segment. The geometrical centroids of clusters/strands were tracked over time to extract their migration parameters.

### Quantification of hydrogel porosity and pore size

DQ-Collagen™ type I from a bovine skin fluorescein conjugate (Thermo Fisher) (final concentration: 25 µg/ml) was added to 2.5, 4 and 6 mg/ml hydrogels to analyze the disposition of the collagen fibers inside the hydrogel^72^. The fluorescence of this reactant was used to analyze the architecture of the 2.5 and 4 mg/ml collagen hydrogels. Due to the high density of the 6 mg/ml collagen gels, the procedure used to visualize this hydrogel was performed using confocal reflectance to improve the quality of the images. A Zeiss LSM 880 confocal microscope equipped with a Plan Apochromat 1.4 63x/1.40 Oil objective was used to visualize the hydrogels.

Confocal cross-section images were sequentially acquired at one point at the center of each microdevice with a constant step size of 0.5 µm. The 3D reconstruction of the z-stack of images of the collagen network was carried out via 3D skeletonization using the FIRE algorithm^73,74^ to obtain a binary stack. From the 3D reconstruction, we quantified the porosity by dividing the total volume of pores between the total volume of the sample being analyzed. Furthermore, we calculated the pore size with another previously described method^75^. Briefly, pore size was determined by obtaining the distribution of the nearest obstacle distances (NODs, defined as the Euclidean distance from a point in the liquid phase to the nearest point in the solid phase).

## Electronic supplementary material

Video 1

Video 2

Video 3

Video 4

Video 5

Video 6

Supplementary information
