# Supplementary material for: From individual to collective 3D cancer dissemination: roles of collagen concentration and TGF-β
Source: Sci Rep. 2018 Aug 24;8:12723. doi: 10.1038/s41598-018-30683-4 (PMC6109049; doi:10.1038/s41598-018-30683-4)
Supplement: Supplementary file 7 — Supplementary information [file 41598_2018_30683_MOESM7_ESM.docx]

Supporting Information

From individual to collective 3D cancer dissemination: roles of collagen concentration and TGF-β

J. Plou*, Y. Juste-Lanas, V. Olivares, C. del Amo, C. Borau, and J.M. García-Aznar*

*Multiscale in Mechanical and Biological Engineering, Aragon Institute of Engineering Research, Department of Mechanical Engineering, University of Zaragoza, 50018 Zaragoza, Spain.*

*Corresponding authors**: jplou@cicbiomagune.es, jmgaraz@unizar.es.

| **Vmean** | | | | | |
| --- | --- | --- | --- | --- | --- |
| **condition 1** | **condition 2** | **lower CI** | **∆ mean** | **upper CI** | **p-value** |
| *2.5* C | *4 C* | 0.0568 | 0.0656 | 0.0744 | 0 |
| *2.5* C | *6 C* | 0.0978 | 0.1066 | 0.1153 | 0 |
| *2.5* C | *2.5 TGF-β* | 0.0328 | 0.0398 | 0.0467 | 0 |
| *2.5* C | *4 TGF-β* | 0.0478 | 0.0557 | 0.0636 | 0 |
| *2.5* C | *6 TGF-β* | 0.0927 | 0.1005 | 0.1082 | 0 |
| *4 C* | *6 C* | 0.0316 | 0.041 | 0.0504 | 0 |
| *4 C* | *2.5 TGF-β* | -0.0336 | -0.0258 | -0.018 | 0 |
| *4 C* | *4 TGF-β* | -0.0185 | -0.0099 | -0.0013 | 0.0141 |
| *4 C* | *6 TGF-β* | 0.0264 | 0.0349 | 0.0434 | 0 |
| *6 C* | *2.5 TGF-β* | -0.0745 | -0.0668 | -0.059 | 0 |
| *6 C* | *4 TGF-β* | -0.0595 | -0.0509 | -0.0423 | 0 |
| *6 C* | *6 TGF-β* | -0.0145 | -0.0061 | 0.0024 | 0.3145 |
| *2.5 TGF-β* | *4 TGF-β* | 0.0091 | 0.0159 | 0.0227 | 0 |
| *2.5 TGF-β* | *6 TGF-β* | 0.0541 | 0.0607 | 0.0673 | 0 |
| *4 TGF-β* | *6 TGF-β* | 0.0372 | 0.0448 | 0.0523 | 0 |
| **Veff** | | | | | |
| **condition 1** | **condition 2** | **lower CI** | **∆ mean** | **upper CI** | **p-value** |
| *2.5* C | *4 C* | 0.014 | 0.0229 | 0.0319 | 0 |
| *2.5* C | *6 C* | 0.019 | 0.0279 | 0.0367 | 0 |
| *2.5* C | *2.5 TGF-β* | 0.0076 | 0.0145 | 0.0215 | 0 |
| *2.5* C | *4 TGF-β* | 0.0108 | 0.0187 | 0.0267 | 0 |
| *2.5* C | *6 TGF-β* | 0.0196 | 0.0275 | 0.0354 | 0 |
| *4 C* | 6 C | -0.0047 | 0.0049 | 0.0145 | 0.6881 |
| *4 C* | *2.5 TGF-β* | -0.0163 | -0.0084 | -0.0005 | 0.0312 |
| *4 C* | *4 TGF-β* | -0.013 | -0.0042 | 0.0046 | 0.7564 |
| *4 C* | *6 TGF-β* | -0.0042 | 0.0046 | 0.0134 | 0.6667 |
| *6 C* | *2.5 TGF-β* | -0.0211 | -0.0133 | -0.0055 | 0 |
| *6 C* | *4 TGF-β* | -0.0178 | -0.0091 | -0.0004 | 0.0331 |
| *6 C* | *6 TGF-β* | -0.009 | -0.0003 | 0.0083 | 1 |
| *2.5 TGF-β* | *4 TGF-β* | -0.0026 | 0.0042 | 0.011 | 0.4854 |
| *2.5 TGF-β* | *6 TGF-β* | 0.0063 | 0.013 | 0.0197 | 0 |
| *4 TGF-β* | *6 TGF-β* | 0.0011 | 0.0088 | 0.0165 | 0.0153 |

Table Sup. 1: pairwise comparison corresponding to mean and effective velocities (Figure 3A).


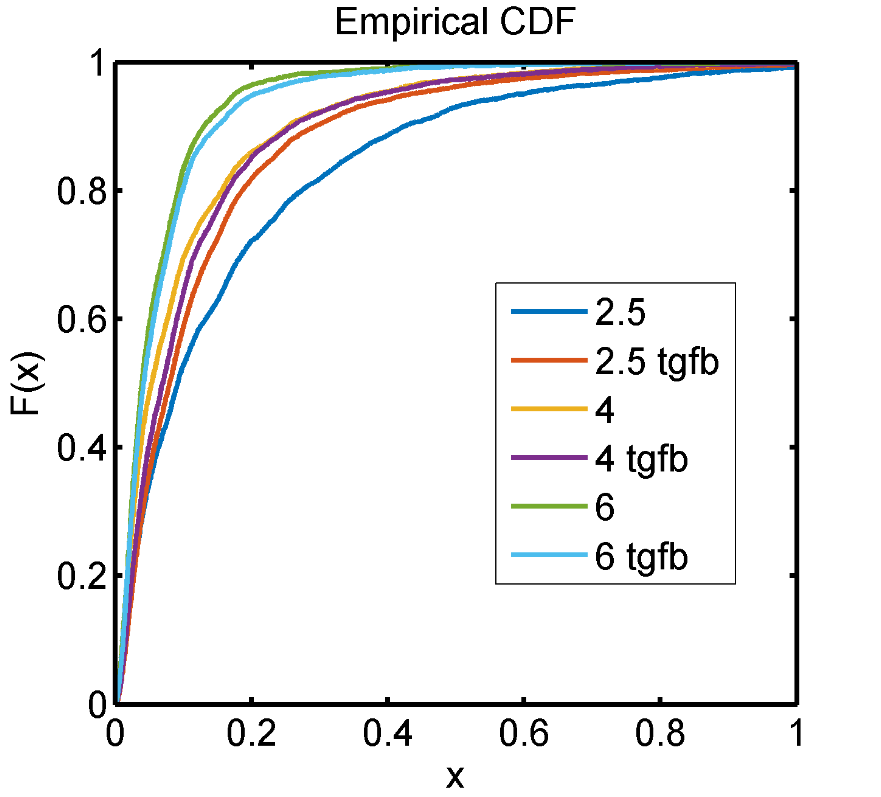


FigSup. 1 Cumulative density function of the mean velocities for each tested condition. Lower curves correspond to higher migration speeds.


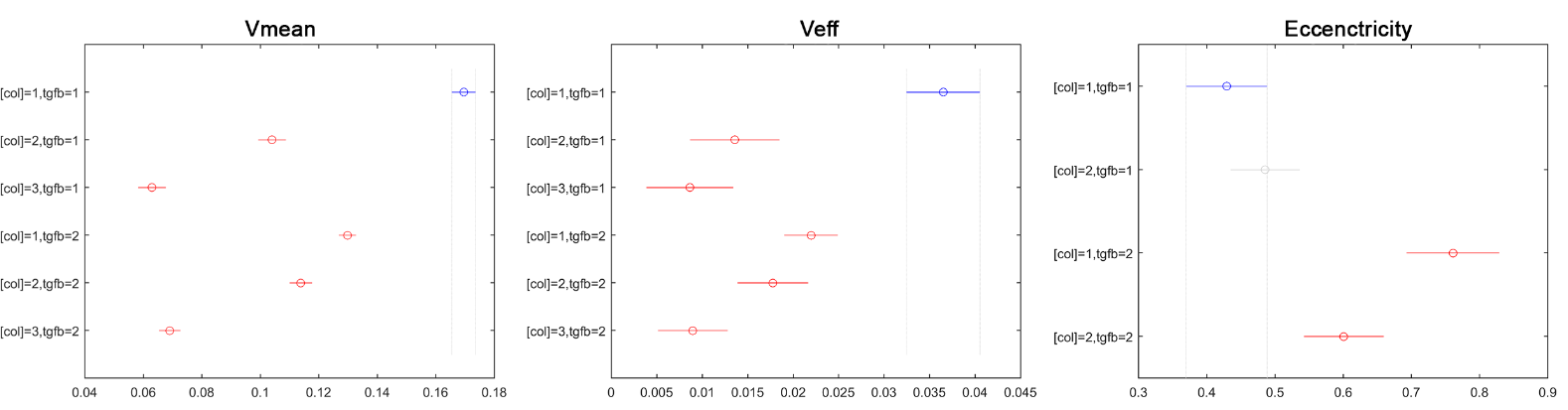


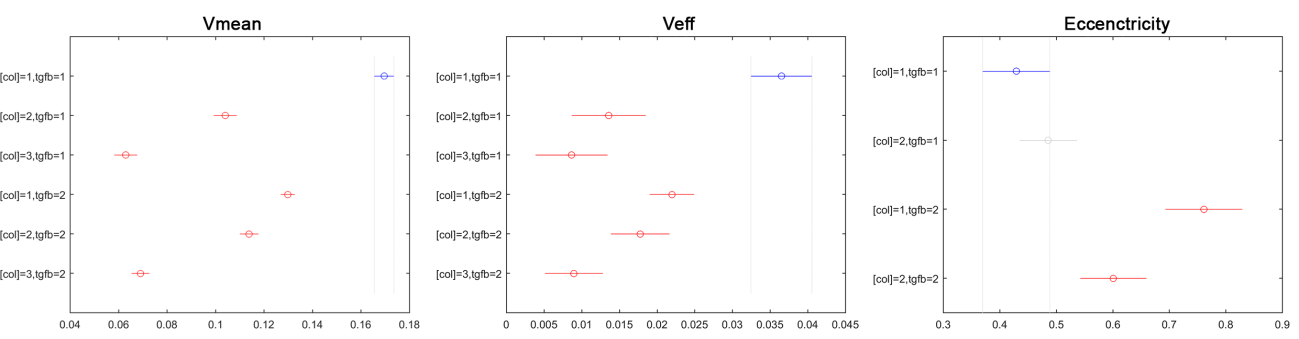


FigSup. 2: Graphical representation of the multivariate analysis of variance performed for Vmean, Veff and Eccentricity (data distributions plotted in Figure 3A). [col] stands for collagen concentration with levels 1,2,3 corresponding to 2.5, 4 and 6 mg/ml respectively; tgfb represents the addition (2) or not (1) of TGF-β. Non-overlapping comparison intervals mean significant statistical differences.


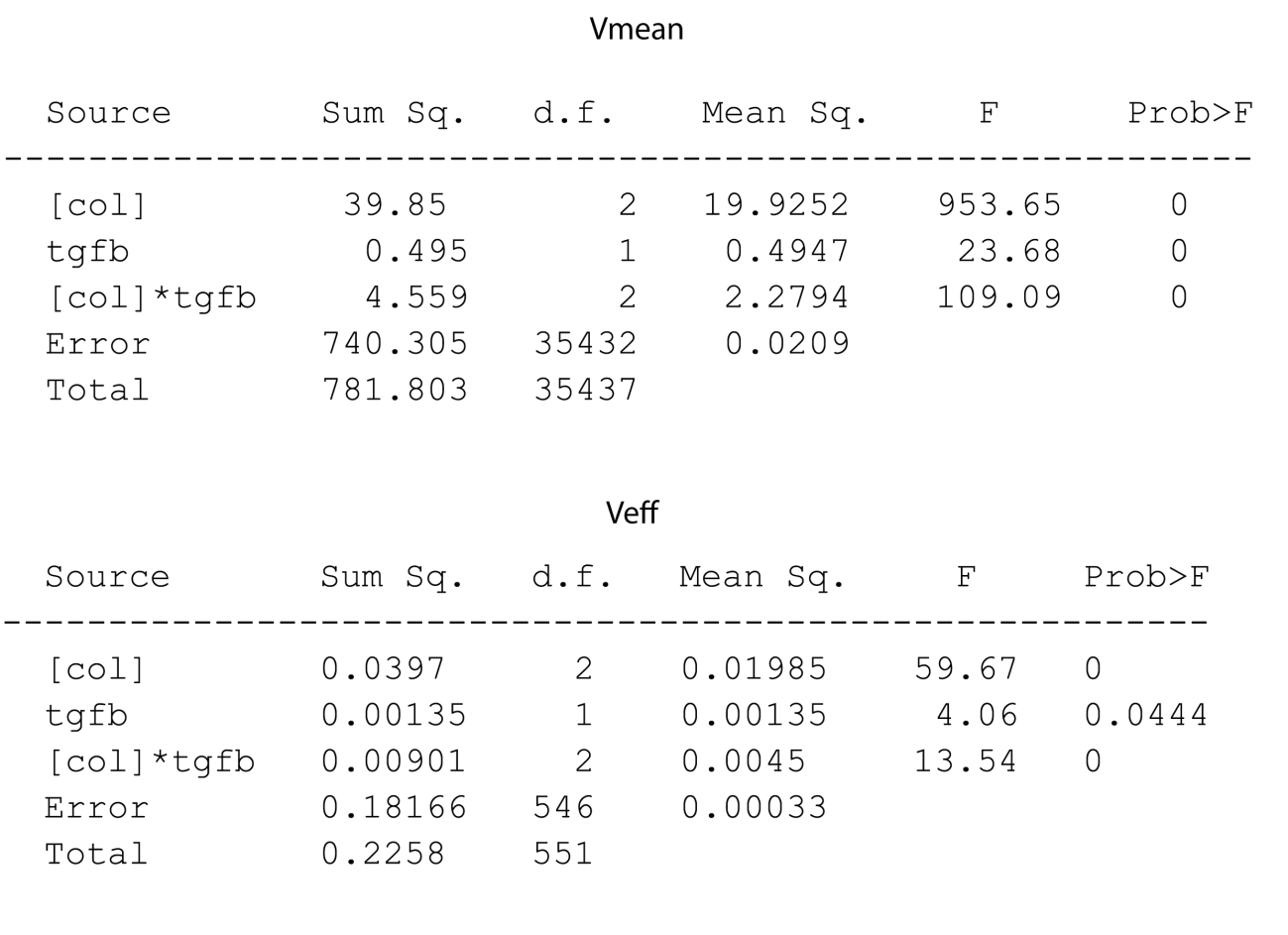


FigSup. 3: Summary tables of the statistical model, including interactions, to test whether the response is the same for all factor levels. [col] stands for collagen concentration with levels 1,2,3 corresponding to 2.5, 4 and 6 mg/ml respectively; tgfb represents the addition (2) or not (1) of TGF-β.

| **Spheroids** | | | | | |
| --- | --- | --- | --- | --- | --- |
| **condition 1** | **condition 2** | **lower CI** | **∆ mean** | **upper CI** | **p-value** |
| *4 C* | *4 TGF-β* | -1.7548 | 11.25 | 24.2548 | 0.0987 |
| *4 C* | *6 TGF-β* | -19.5048 | -6.5 | 6.5048 | 0.4759 |
| *6 C* | *4 TGF-β* | 5.4952 | 18.5 | 31.5048 | 0.0056 |
| *6 C* | *6 TGF-β* | -12.2548 | 0.75 | 13.7548 | 0.9981 |
| *4 TGF-β* | *6 TGF-β* | -30.7548 | -17.75 | -4.7452 | 0.0075 |
| **Strands** | | | | | |
| **condition 1** | **condition 2** | **lower CI** | **∆ mean** | **upper CI** | **p-value** |
| *4 C* | *6 C* | -4.3126 | 1.25 | 6.8126 | 0.9074 |
| *4 C* | *4 TGF-β* | -11.3126 | -5.75 | -0.1874 | 0.042 |
| *4 C* | *6 TGF-β* | -11.3126 | -5.75 | -0.1874 | 0.042 |
| *6 C* | *4 TGF-β* | -12.5626 | -7 | -1.4374 | 0.0131 |
| *6 C* | *6 TGF-β* | -12.5626 | -7 | -1.4374 | 0.0131 |
| *4 TGF-β* | *6 TGF-β* | -5.5626 | 0 | 5.5626 | 1 |

Table Sup. 2: pairwise comparison corresponding to number of spheroids and strands (Figure 4B).


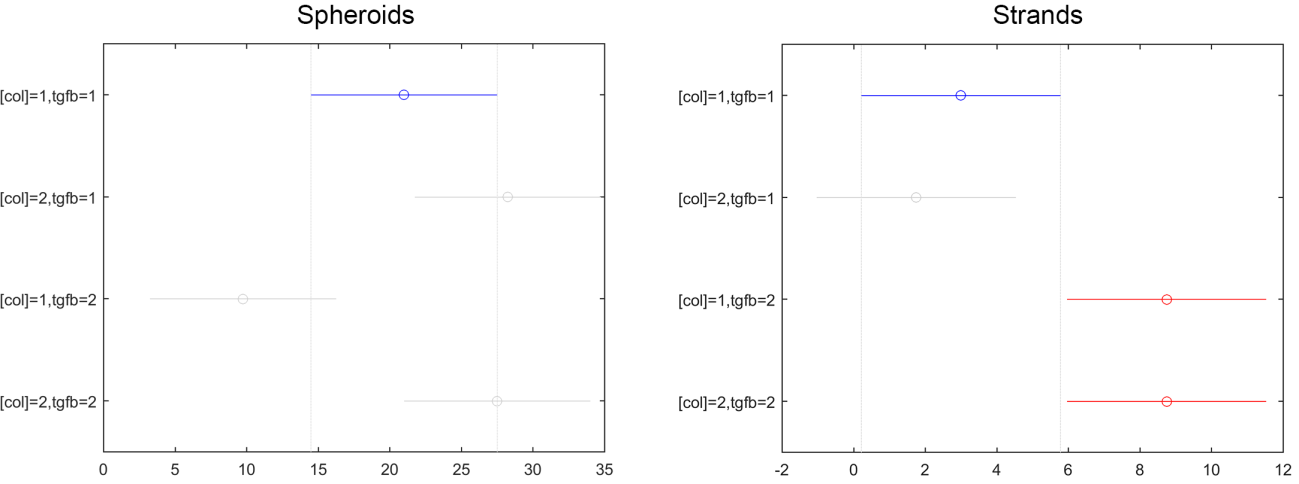


FigSup. 4: Graphical representation of the multivariate analysis of variance performed for the number of spheroids and strands in different conditions (data distributions plotted in Figure 4B). [col] stands for collagen concentration with levels 1,2 corresponding to 4 and 6 mg/ml respectively; tgfb represents the addition (2) or not (1) of TGF-β. Non-overlapping comparison intervals mean significant statistical differences.


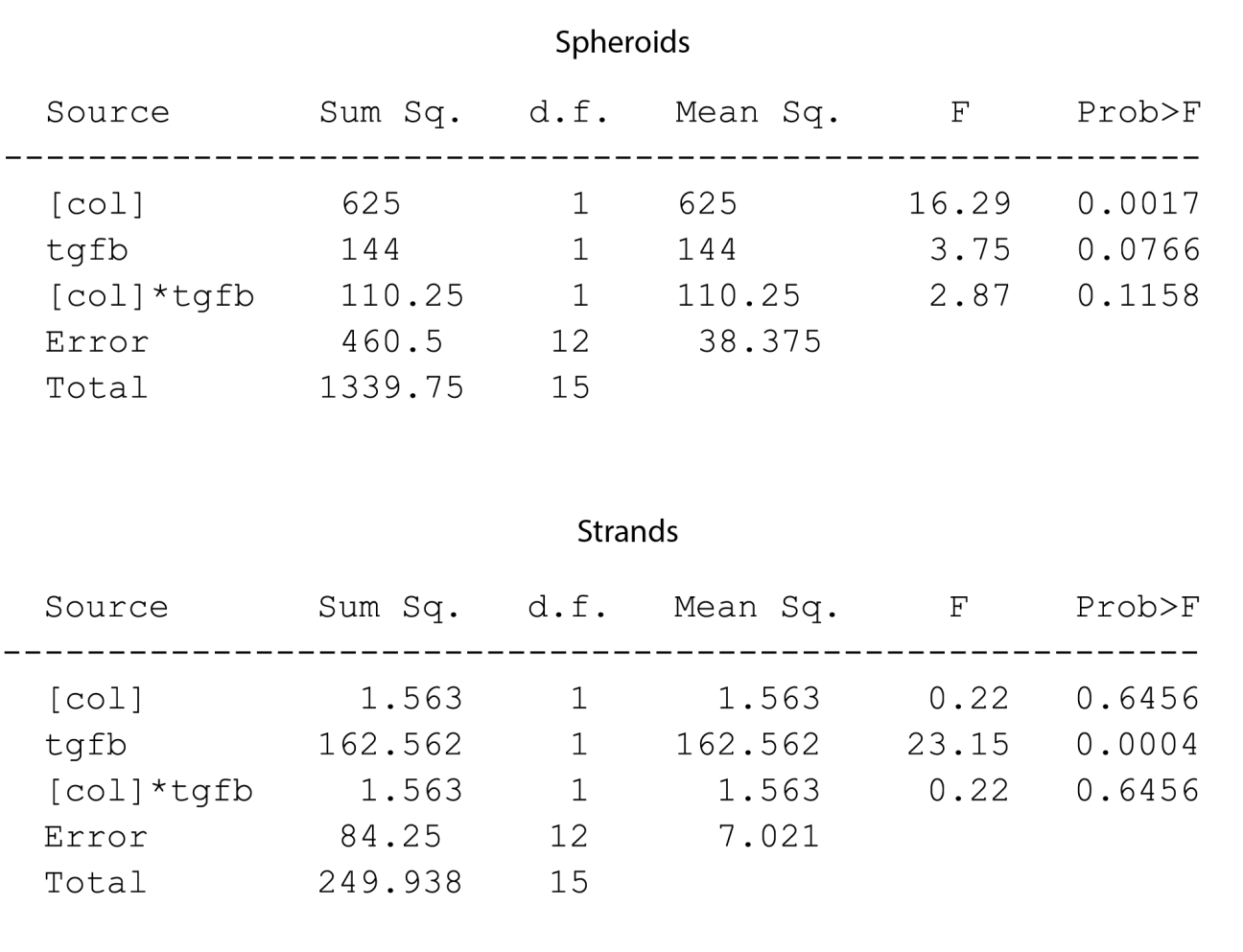


FigSup. 5: Summary tables of the statistical model, including interactions, to test whether the response is the same for all factor levels. [col] stands for collagen concentration with levels 1,2 corresponding to 4 and 6 mg/ml respectively; tgfb represents the addition (2) or not (1) of TGF-β.


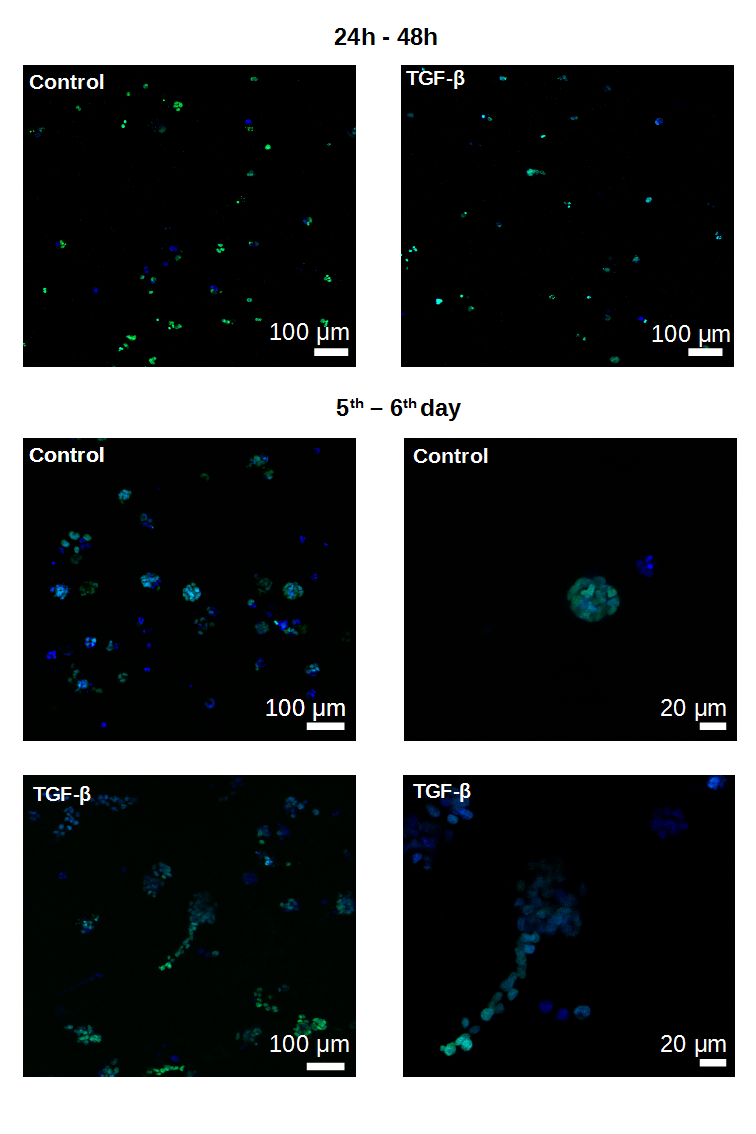


FigSup. 6 Fluorescent confocal images of H1299 stained with DAPI (blue) and a cell proliferative marker, EdU (green) under diverse times (48h and 6thday) and conditions, control and TGF-β stimulation (100x and 250x magnification). Cells are able to proliferate throughout the entire 7 days experiment and no significant differences are qualitatively appreciated among control and TGF-β conditions at early and longer stages of the 3D cell culture. Specifically, spheroids and strands show both a high percentage of proliferative cells.


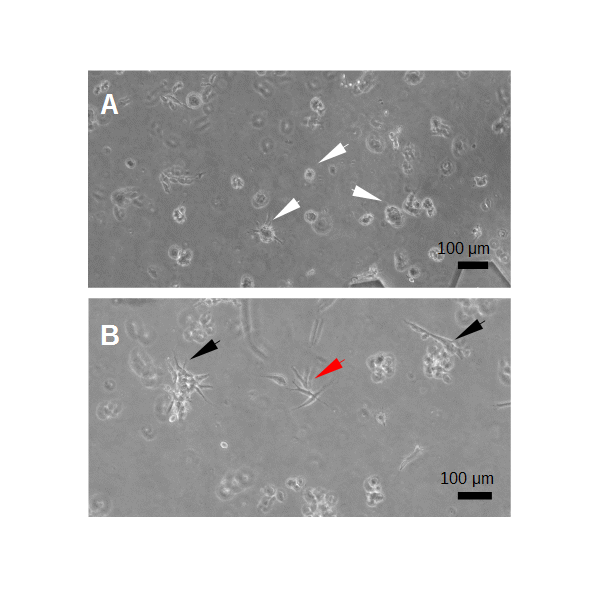


FigSup. 7 Bright-field images of MBA-MB-231 cell line taken after 7 days (A) and TGF-β treatment (10ng/mL) (B) in 6 mg/mL collagen matrices. Strand-like morphologies (eccentricity > 0.8) are indicated with black arrows. Spheroids (eccentricity < 0.8) are indicated with white arrows. Elongated individual cell are indicated with red arrows. Scale bar is 100 μm for both panels
